# Effectiveness of a Fully Online Scientific Research Works Peer Support Group Model for Research Capacity Building Through Conducting Systematic Reviews Among Health Care Professionals: Retrospective Cohort Studies

**DOI:** 10.2196/78862

**Published:** 2025-10-02

**Authors:** Yuki Kataoka, Ryuhei So, Masahiro Banno, Yasushi Tsujimoto, SRWS-PSG Mentors

**Affiliations:** 1 Scientific Research WorkS Peer Support Group, 12-12 Osaka Ekimae Dai-2 Building 1-2-2 Umeda, Kita-ku, Osaka, 5300001, Japan, 81 757016111; 2 Center for Postgraduate Clinical Training and Career Development, Nagoya University Hospital, Aichi, Japan; 3 Center for Medical Education, Graduate School of Medicine, Nagoya University, Aichi, Japan; 4 Department of Internal Medicine, Kyoto Min-iren Asukai Hospital, Kyoto, Japan; 5 Department of Healthcare Epidemiology, Kyoto University Graduate School of Medicine/School of Public Health, Kyoto, Japan; 6 Department of International and Community Oral Health, Tohoku University Graduate School of Dentistry, Miyagi, Japan; 7 Department of Psychiatry, Okayama Psychiatric Medical Center, Okayama, Japan; 8 CureApp, Inc., Tokyo, Japan; 9 Department of Psychiatry and Neurology, Seichiryo Hospital, Nagoya, Japan; 10 Oku Internal Medicine Clinic, Osaka, Japan; 11 Department of Health Promotion and Human Behavior, Kyoto University Graduate School of Medicine/School of Public Health, Kyoto, Japan

**Keywords:** research capacity building, online education, systematic review, peer support group, health care professionals, mentoring, e-learning

## Abstract

**Background:**

Research capacity building (RCB) among health care professionals remains limited, particularly for those working outside academic institutions. Japan is experiencing a decline in original clinical research due to insufficient RCB infrastructure. Our previous hospital-based workshops were effective but faced geographical and sustainability constraints. We developed a fully online Scientific Research Works Peer Support Group (SRWS-PSG) model that addresses geographical and time-bound constraints and establishes a sustainable economic model. Mentees use online materials, receive support from mentors via a communication platform after formulating their research question, and transition into mentors upon publication.

**Objective:**

We evaluated whether our model’s theoretical benefits translated into actual program effectiveness in RCB among health care professionals.

**Methods:**

We conducted a retrospective cohort study of health care professionals who participated in the SRWS-PSG program between September 2019 and January 2025. Mentees progressed through a structured modular curriculum covering systematic review methodology, from protocol development to manuscript preparation, with personalized mentoring support. We evaluated manuscript submission, program discontinuation, promotion to a mentor status, and mentor response time. We collected data from program records and chat logs. Manuscript submission was defined as mentor-confirmed submission of a systematic review manuscript to a peer-reviewed journal. Program discontinuation referred to formal withdrawal before manuscript submission. Mentor promotion was defined as acceptance of an invitation to serve as a junior mentor after manuscript submission. Mentor response time was the elapsed time from a mentee’s question in the chat to the first reply by an assigned mentor.

**Results:**

Of 85 mentees analyzed, 31 (36.5%) held academic degrees (PhD or MPH), and 68 (80%) were medical doctors. During a median follow-up of 10 months, 51 (60%) submitted manuscripts and 46 (90%) became mentors. Ten mentees (12%) discontinued the program. The median mentor response time was 0.8 hours, with 90% responding within 24 hours.

**Conclusions:**

A majority of participants of SRWS-PSG submitted manuscripts. This fully online RCB program might address geographical barriers and provides an adaptable approach for RCB across diverse health care contexts.

## Introduction

Research capacity building (RCB) among health care professionals is essential for addressing clinical challenges and improving health care quality. Cooke’s RCB framework [1] comprises four structural levels (individual, team, organizational, and network/supra-organizational) and six principles (developing skills and confidence, supporting linkages and partnerships, ensuring research close to practice, enabling appropriate dissemination, investing in infrastructure, and building sustainability and continuity). A systematic review showed that RCB strategies are interlinked and interdependent, requiring implementation through an integrated “whole of system” approach with commitment and support from all levels of leadership and management [2]. Similarly, a scoping review found that these programs are typically multifaceted, often incorporating experiential learning and mentoring, but evaluations predominantly focus on lower levels of Kirkpatrick’s educational outcomes typology (eg, participant satisfaction, improved knowledge, and confidence), with few assessing objective milestones (eg, protocol completion or manuscript preparation) or broader organizational and practice impacts. The authors conclude that rigorous evaluations are needed, considering long-term outcomes and translation into clinical practice, to better bridge the research-practice gap [3].

RCB programs for practicing health care professionals outside academic institutions remain limited [2]. In Japan, the decline in original clinical research is a recognized issue [4,5]. This is largely due to insufficient RCB infrastructure, a problem that affects both academic and non-academic settings [6,7]. Like in other countries, there are often no clear expectations or structured incentives for clinicians to engage in research [8]. The situation is worsened by systemic funding shortages in academia [9]. Many Japanese clinicians are therefore motivated not by the prospect of academic promotion, but by a sense of professional responsibility—or “rectitude,” a concept deeply rooted in the traditional Japanese code of conduct [10]—to address clinical questions that arise from their practice. Our program was developed to support these grass-roots community healthcare professionals, who are motivated to address their research questions [11]. The program aimed to build research capacity from the bottom up. Between 2014 and 2017, we conducted a structured in-person scholarly productive model workshop for hospital-based health care professionals. This workshop developed a curriculum and achieved academic publications from participants through team-based systematic review projects. However, this hospital-based implementation faced two constraints: limited dissemination capacity and unsustainable dependence on voluntary mentor work. In 2019, we transformed the workshop into a fully online peer support program (Scientific Research Works Peer Support Group [SRWS-PSG]) with participant fees supporting mentor compensation. This redesign addressed geographical and time-bound barriers, strengthened cross-institutional partnerships, and established a sustainable economic model for ongoing RCB activities. The program features a comprehensive curriculum with over 140 modules covering all aspects of systematic review methodology. Mentees progress through self-paced online materials and receive feedback from mentors on their protocols, manuscripts, and any questions about the systematic review process via an internet communication platform, eventually transitioning into mentors upon publication.

We previously developed a structured, in-person scholarly productive model workshop for hospital-based health care staff between 2014 and 2017 [11]. This workshop developed a curriculum and achieved academic publications from participants through team-based systematic review projects. However, this hospital-based implementation faced two constraints: limited dissemination capacity and unsustainable dependence on voluntary mentor work. In 2019, we transformed the workshop into a fully online peer support program (SRWS-PSG) with participant fees supporting mentor compensation. This redesign addressed geographical and time-bound barriers, strengthened cross-institutional partnerships, and established a sustainable economic model for ongoing RCB activities. The program features a comprehensive curriculum with over 140 modules covering all aspects of systematic review methodology. Mentees progress through self-paced online materials and receive feedback from mentors on their protocols, manuscripts, and any questions about the systematic review process via an internet communication platform, eventually transitioning into mentors upon publication.

While the need for RCB for health care professionals is clear, scalable and sustainable programs remain scarce, particularly for those outside academic institutions in Japan. This study, therefore, aimed to evaluate the effectiveness of our fully online and economically self-sustaining SRWS-PSG model. Our research question was “How effective is this program in enabling health care professionals to complete and submit systematic reviews?” To address this, we conducted a retrospective cohort study analyzing data from participants enrolled between 2019 and 2025. We hypothesized that this peer-supported, online model would demonstrate a high proportion of manuscript submission and program continuation, validating its utility as a scalable RCB solution. The findings are intended to inform medical educators, hospital administrators, and health policymakers seeking to develop and implement effective RCB programs.

## Methods

### Study Design

We conducted a retrospective cohort study of health care professionals who participated in the SRWS-PSG program between September 2019 and January 2025. The SRWS-PSG is designed specifically to support health care professionals from any discipline in conducting systematic reviews as their primary research output. The SRWS-PSG program is now operated by the SRWS-PSG, a non-profit general incorporated association registered in Japan (corporate Number: 6120005024588). The mission is to increase clinical research in Japan. The members consist of health care professionals, and it is funded through membership and mentorship fees. The program provides a structured, modular curriculum covering the entire systematic review process, from protocol development to manuscript submission. Learning is facilitated through self-paced online materials and personalized, responsive mentorship via a communication platform. We applied Cooke’s RCB framework [1] for the methodology and structured our findings. To report this article, we followed the Strengthening the Reporting of Observational Studies in Epidemiology (STROBE) statement [12].

### Setting

This study was conducted in a fully online environment. We recruited participants through social media, websites, academic conferences, and referrals from previous participants. The primary registration portal was CAMPFIRE, a Japanese crowdfunding platform [13]. We collected data from Slack chat logs, program participation records, and manuscript submission status. Occupations and academic degrees came from registration forms. The data cut-off was February 2025.

### Participants

**Figure 1.**
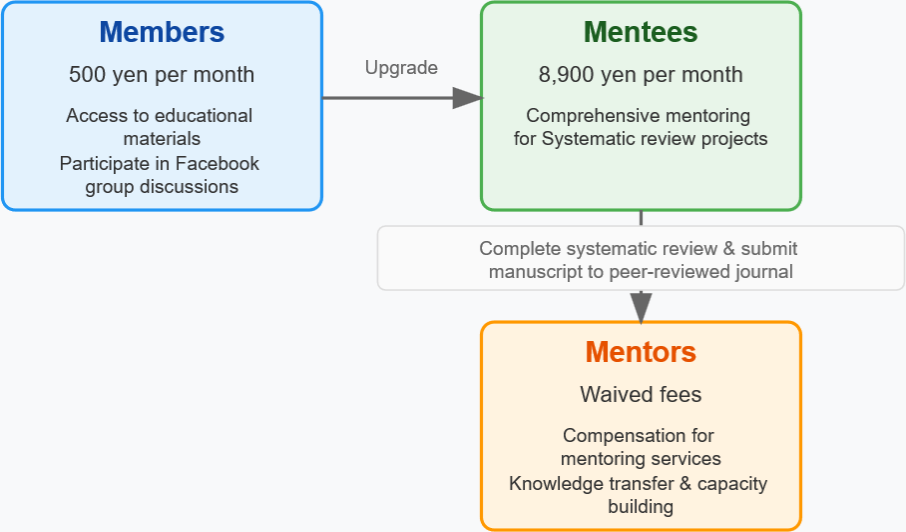
The Scientific Research Works Peer Support Group (SRWS-PSG) membership structure.

As of 2025, the SRWS-PSG uses a tiered membership structure with different levels of engagement and fee schedules. Entry-level “members” pay 500 yen (3‐4 US$) per month to access educational materials and participate in research-related discussions through a Facebook group. “Mentees” pay 8900 yen (around 60 US$) per month for comprehensive mentoring support throughout their systematic review projects. Mentees who submit their manuscript to a peer-reviewed journal are invited to become mentors—with waived fees and compensation—if their two assigned mentors assess them as suitable for the role based on their aptitude for chat-based communication (Figure 1).

In this study, we analyzed mentees who were health care professionals from various disciplines including physicians, nurses, pharmacists, physical therapists, clinical psychologists, and registered dietitians. All mentees could communicate online, had research questions suitable for systematic review topics, and agreed to participate. None had conducted a systematic review as the lead author. We excluded those withdrew before program initiation or did not complete initial training. The cumulative incidences of manuscript submission and discontinuation of the program, stratified by university affiliation, are plotted with 95% CIs. Shaded areas represent the 95% CIs.

### Curriculum

The SRWS-PSG curriculum uses outcome-based learning objectives to enable health care professionals to conduct systematic reviews and publish in peer-reviewed journals [14]. Objectives include formulating research questions, developing literature search strategies, performing critical appraisal, synthesizing evidence, evaluating certainty of evidence, and producing academic manuscripts meeting international publication standards. The curriculum follows a modular progression aligned with the systematic review process, from orientation modules through protocol development, execution, analysis, and manuscript preparation (Multimedia Appendix 1). Training materials are in Japanese. To become familiar with English academic writing, mentees without English writing experience critically appraised an article and submitted a letter to the editor before formulating their research question. The program incorporates methodological updates including the latest Cochrane Handbook [15] and Risk of Bias 2 tool [16] to ensure current best practices.

The instructional methodology combines asynchronous self-paced learning with responsive mentoring support. Mentees first view 10 to 30 minutes of video content explaining systematic review concepts and methodologies. Mentees then draft specific sections of their research protocols—structured tasks with concrete deliverables. Each mentee receives personalized feedback from two assigned mentors—a junior and a senior mentor—through Slack, a business-oriented chat application [17]. Mentors serve as co-authors on the mentee’s systematic review project. Junior mentors are recent program graduates, who are promoted to senior status after guiding three mentees to publication. Mentors respond within 24 hours, while mentees follow a “15-minute rule” (a structured guideline that requires mentees to attempt solving methodological or procedural challenges independently for at least 15 min before posting questions to mentors, designed to promote self-reliance, enhance problem-solving skills, and ensure efficient use of mentoring resources). Optional synchronous online meetings occur upon mentee request.

To enhance learning through practical application, members actively contribute to other mentees’ projects. This approach leverages the systematic review methodology’s requirement for independent dual assessment of article screening, data extraction, and risk of bias evaluation [18]. This collaborative structure enhances learning through practical application.

### Outcomes

#### Overview

We selected four outcomes based on data available from our existing program records. These outcomes partially reflect key dimensions of Cooke’s RCB framework [1]. Manuscript submission was chosen as an indicator of skill development and confidence building, representing participants’ ability to complete a systematic review project. Program discontinuation was measured to assess individual-level sustainability and continuity. Promotion to mentor status following manuscript submission was evaluated as a marker of organizational-level sustainability and continuity. Mentor response time was analyzed as a proxy for infrastructure investment and team support quality. The outcomes were described as given below.

#### Manuscript Submission

Manuscript submission was defined as the time from mentor approval of the mentee’s research question to start the protocol to the submission of systematic review manuscripts to academic journals. Mentees self-reported manuscript submission with mentor confirmation.

#### Program Discontinuation

Discontinuation was defined as when a mentee formally notified the program of their withdrawal, leading to the cessation of monthly program fee payments prior to manuscript submission.

#### Promotion to a Mentor

Promotion to mentor status was evaluated by determining the proportion of manuscript-submitting mentees who accepted the invitation to become a junior mentor after being assessed as suitable by their assigned mentors. This transition was documented in program administration records.

#### Mentor Response Time

The mentor response time was defined as the time from a mentee posting a question in a designated Slack channel to the first response from any assigned mentor in the same channel. Data were extracted from Slack chat logs.

### Study Size

We included all mentees during the program operation period, without formal sample size calculation. This size was similar to that in other research capacity development program evaluation studies [19,20].

### Statistical Analysis

We used descriptive statistics to summarize participant characteristics, continuation rate, and mentor response time. Manuscript submission and publication were analyzed as time-to-event outcomes. We estimated the cumulative incidence functions for both submission and publication using the Aalen-Johansen estimator [21]. Discontinuation from the program was a competing risk. Competing risk occurs when an event (manuscript submission) cannot occur because another event (discontinuation) happens first. We censored participants still active without an event at data cut-off. For subgroup analyses, we performed univariable Fine-Gray subdistribution hazard models to calculate subdistribution hazard ratios (sdHR) with 95% CIs for academic degree, profession, and university affiliation. We performed analyses using Python (version 3.11.4; Python Software Foundation) and R (version 4.3.2; R Foundation for Statistical Computing). Python packages included *lifelines* (version 0.30.0) and *scikit-survival* (version 0.24.1). R packages included *cmprsk* (version 2.2.12).

### Ethical Considerations

The Ethics Committee of Kyoto Min-iren Asukai Hospital approved the study protocol (Approval number: 202502‐3) and waived the need for individual informed consent. The waiver was granted because this retrospective study analyzed routinely collected clinical data without any direct contact or intervention, posed no more than minimal risk to participants, and obtaining consent from all eligible patients would have been impracticable. All procedures complied with the institutional ethics standards and the Declaration of Helsinki. To protect privacy and confidentiality, the analytic dataset was deidentified before analysis: direct identifiers were removed. Study data were kept on access-controlled servers.

## Results

### Participants

During the study period, 118 individuals registered. After excluding 33 participants (30 who failed initial training for formulating research questions, and 3 who did not start), 85 mentees entered the final analysis. Of 85 mentees, 31 (36.5%) held academic degrees (PhD: 27, MPH: 2, PhD and MPH: 2), and 44 (51.8%) were working at university-affiliated hospitals. Sixty-eight (80%) were medical doctors, and 17 (20%) represented other health care professions: physical therapists (n=8), pharmacists (n=3), clinical psychologists (n=2), nurses (n=1), registered dietitians (n=1), dentists (n=1), and unspecified (n=1). No mentees had missing follow-up data.

### Outcomes

**Figure 2.**
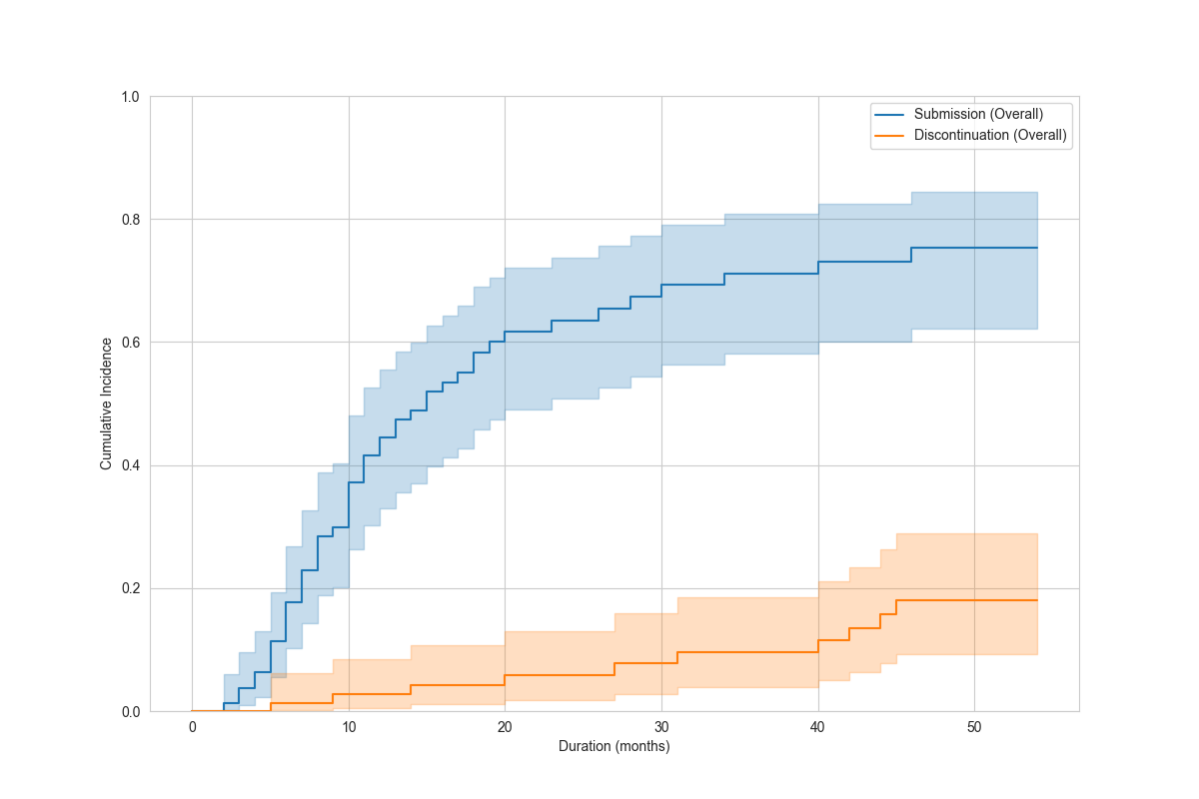
Cumulative incidence of manuscript submission and discontinuation of the program are plotted with 95% CIs. Shaded areas represent the 95% CIs.

**Figure 3.**
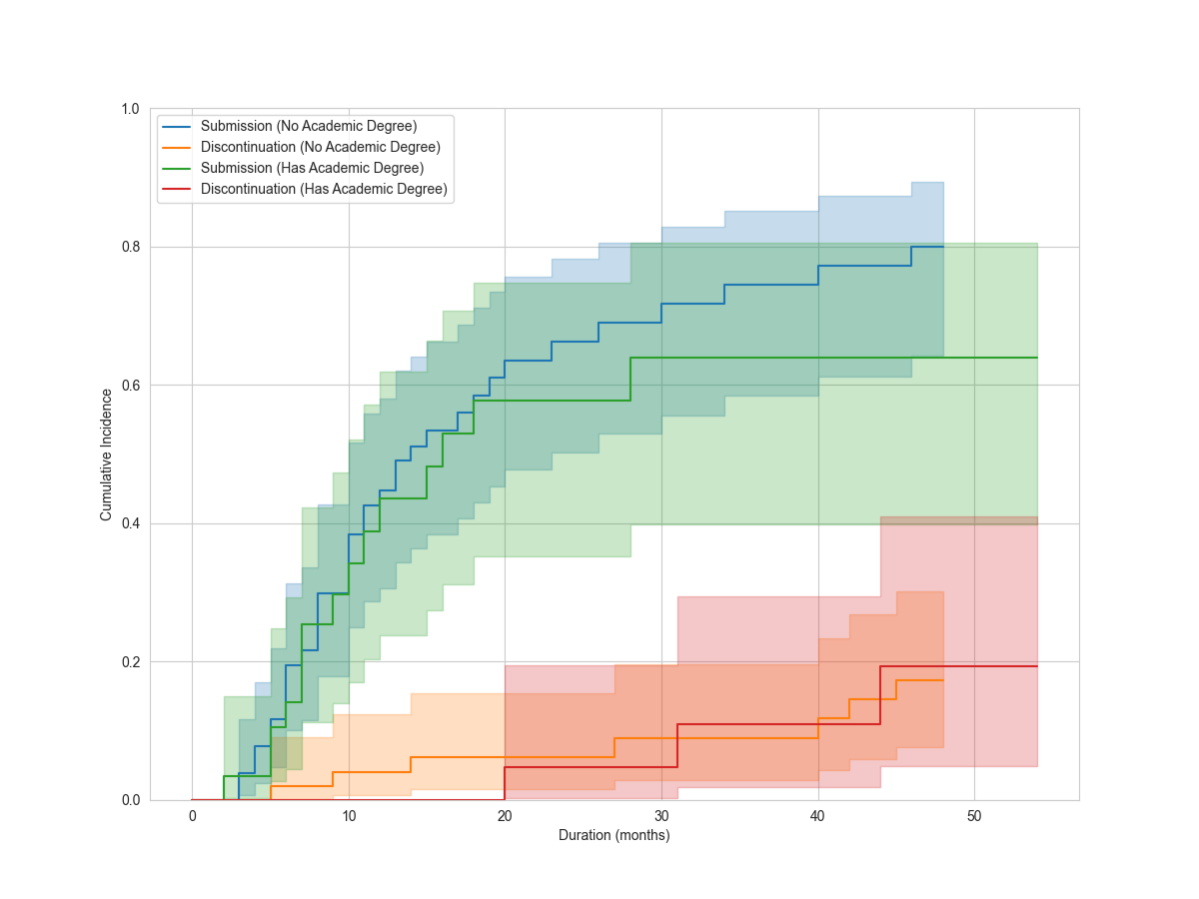
Cumulative incidence of manuscript submission and discontinuation of the program, stratified by the presence or absence of an academic degree (PhD or MPH), are plotted with 95% CIs. Shaded areas represent the 95% CIs.

During a median follow-up of 10 months (range, 0‐54 months), 51 mentees (60%) submitted manuscripts. Forty-six (90%) became mentors. Ten (12%) discontinued. Discontinuation reasons included work commitments (n=4), workplace change (n=2), unclear (n=2), personal reasons (n=1), and competing systematic review publication (n=1). Twenty-four mentees continue projects. Cumulative manuscript submission reached 75.2% (95% CI 62.1%‐84.3%), and cumulative discontinuation was 18% (95% CI 9.3%‐28.9%) at the end of follow-up (Figure 2). At 12 months, manuscript submission was 42.9% (95% CI 31.4%‐53.9%) and discontinuation was 2.7% (95% CI 0.5%‐8.5%). At 24 months, manuscript submission was 63.3% (95% CI 50.6%‐73.6%) and discontinuation was 5.8% (95% CI 1.9%‐13.1%). In univariable analyses, the sdHR for manuscript submission was 0.78 (95% CI 0.43‐1.43) for academic degree, 1.00 (95% CI 0.53‐1.86) for profession, and 1.31 (95% CI 0.77‐2.24) for university affiliation (Figures3^5^). Of 51 submissions, 50 appeared in English peer-reviewed journals. One submission remains under peer review. Submissions included 32 pairwise meta-analyses, 7 scoping reviews, 4 network meta-analyses, 2 prognostic studies, 2 diagnostic test accuracy studies, and 3 other study types (Multimedia Appendix 2). All published articles were indexed in the Web of Science at the time of publication. Journal impact factors were available for all except *The Annals of Translational Medicine*, which was indexed in the Web of Science and PubMed at submission but later excluded from the Web of Science. Citation counts, disregarding time since publication, had a median of 6 (range 0‐60) as of Aug 1, 2025. Twelve articles (24%) appeared in journals requiring article processing charges. Analysis of 58,952 messages showed a median mentor response time of 0.8 hours (IQR 0.1‐6.1), with 90% responding within 24 hours.

**Figure 4.**
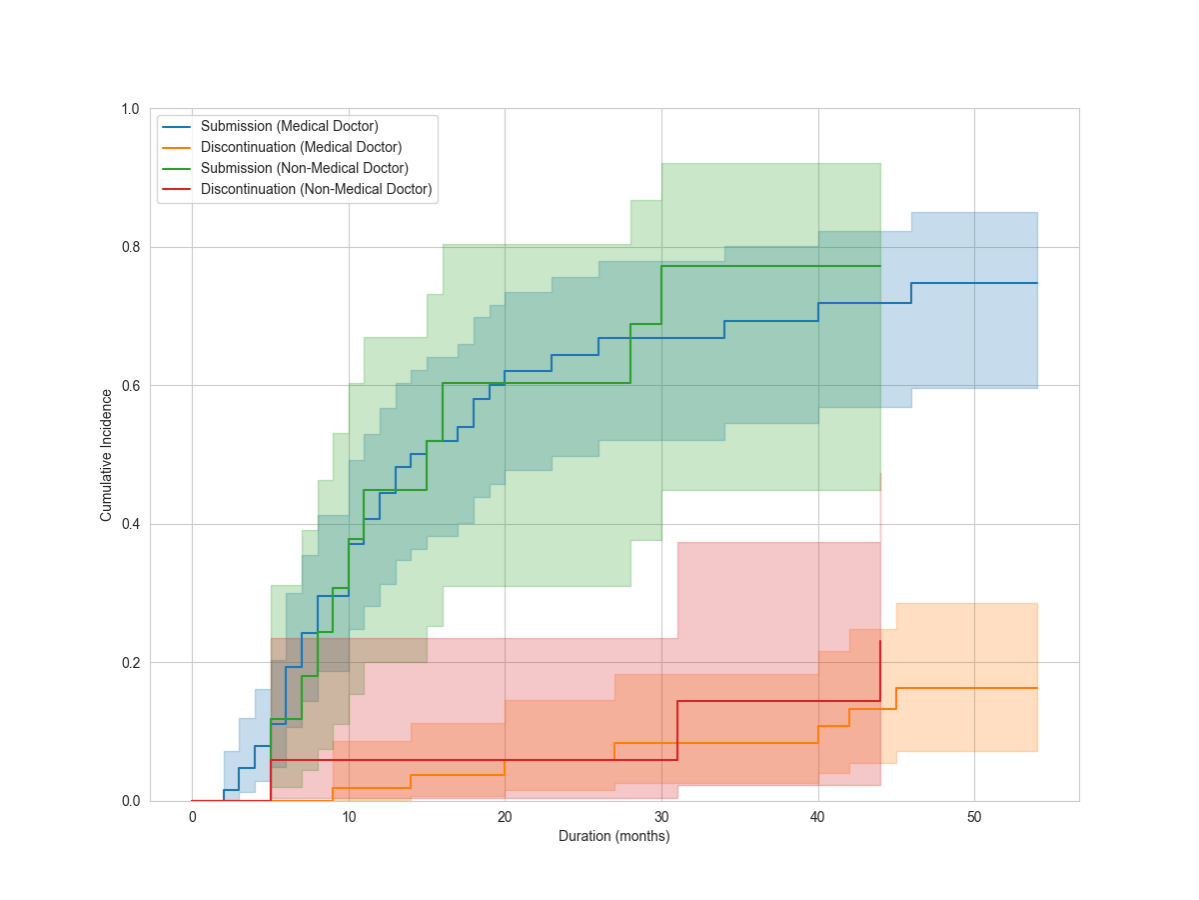
Cumulative incidences of manuscript submission and discontinuation of the program, stratified by profession (medical doctor or non-medical doctor), are plotted with 95% CIs. Shaded areas represent the 95% CIs.

**Figure 5.**
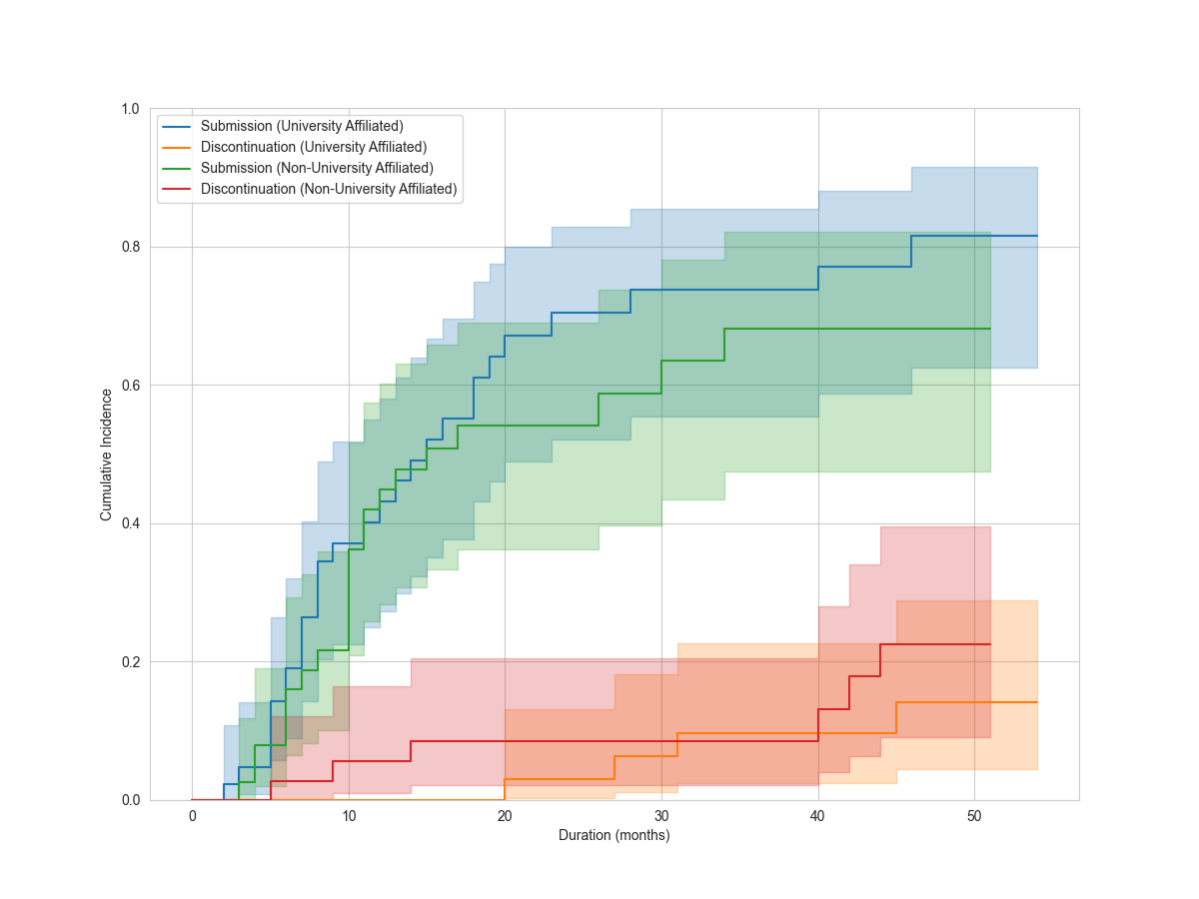
Cumulative incidences of manuscript submission and discontinuation of the program, stratified by university affiliation, are plotted with 95% CIs. Shaded areas represent the 95% CIs.

## Discussion

### Principal Results

The principal finding is that our fully online, peer-supported RCB model might contribute to actual research outputs for health care professionals. Specifically, cumulative manuscript submission reached 75.2% at the end of follow-up, while cumulative discontinuation remained at 18%. The publications appeared in journals indexed in the Web of Science, with a median citation count of 6 (range 0‐60), indicating initial uptake. The rapid mentor responsiveness (median 0.8 hours) further contributed to program effectiveness. Ninety percent of mentees who submitted manuscripts transitioned to mentors. These quantitative outcomes provide a foundation for the subsequent discussion of the program’s effectiveness through the lens of Cooke’s RCB framework [1].

While we did not set specific a priori cut-offs for outcomes, we consider the observed outcomes meaningful. Of note, we view discontinuation as a particularly critical metric because it directly reflects program adequacy—participants who leave the program cannot achieve the primary outcome of manuscript submission. The reasons for discontinuation (work commitments, workplace changes, personal reasons, and competing publications) were all external factors beyond the scope of SRWS-PSG’s organizational support, suggesting that the program’s support structure itself was adequate.

### Implications of Findings

Research-novice medical professionals were able to participate in the SRWS-PSG program. Our subgroup analyses demonstrated that manuscript submission rates were similar regardless of participants’ academic degrees, professional backgrounds, or institutional affiliations. The program’s fully online format addresses geographical and temporal constraints, enabling flexible participation around clinical duties. Rapid mentor feedback provides continuous support for novices navigating the systematic review process. This accessibility suggests that the SRWS-PSG model might expand RCB opportunities beyond traditional academic boundaries.

Hospital administrators seeking to foster their medical professionals’ research competency could provide protected time for SRWS-PSG participation. The primary reasons for program discontinuation were external factors such as work commitments and workplace changes rather than program inadequacy. Institutional support through protected time could address these barriers and enhance completion rates [6]. The program’s modest monthly fee represents a cost-effective investment for institutions.

The SRWS-PSG model’s features suggest broad applicability across diverse global contexts, including low- and middle-income countries. The fully online format eliminates geographical barriers and infrastructure requirements, while the focus on systematic reviews—requiring only internet access without expensive equipment or data collection costs—makes research accessible in resource-limited settings. The self-sustaining economic model through participant fees offers an alternative to scarce grant funding. While implementation would require translation and fee adjustment to local contexts, the core elements—peer support, standardized methodology following international guidelines, and progression from mentee to mentor—address universal challenges in RCB regardless of economic or cultural setting.

### Comparison to the Literature

Our findings extend previous RCB evaluations in several ways. The SRWS-PSG model’s continuous feedback approach shares elements with intensive programs like writing boot camps [22] but delivers them in a sustained, online format throughout the entire systematic review process. This contrasts traditional time-limited workshops. Our approach shares goals with the Rural Research Capacity Building Program in Australia [20] but operates entirely online.

Mentees formulated research questions directly relevant to their clinical environments, bridging research and practice in ways that promote Boyer’s scholarship concept of “integration” and “application” [23]. This contrasts with academic programs where research topics may be driven by funding or institutional priorities rather than clinical needs.

The self-supporting economic model offers a sustainable alternative to grant-dependent programs. Mentee fees fund mentor compensation and administrative costs. This enables 5 years of operation without external funding. Many RCB programs require institutional or funding support [2]. This approach echoes Japan’s Edo period tradition of community-based education. Community mathematics teachers (“Wasan-ka”) sustained themselves through teaching fees. This grass-roots system enabled people from all social classes to publish their mathematical problems at shrines as open access articles (“Sangaku”) [24]. SRWS-PSG functions as a traditional citizen science model, maintaining economic sustainability through community participation rather than institutional supports.

### Limitations and Future Directions

This study has several limitations. First, this retrospective observational study without a control group cannot establish causality. Although continuous program improvements pose a challenge for evaluation, a rigorously designed well-powered randomized controlled trial is the necessary next step to definitively assess the efficacy. Second, our metrics emphasized outputs such as submission but did not directly evaluate some dimensions of Cooke’s framework. Specifically, we did not assess changes in individual research skills (including systematic review methodology competency beyond evidence-based medicine competency [25]), self-reported confidence, or participant motivation levels. We also could not evaluate the quality of mentor-mentee relationships, organizational-level support, and infrastructure investments, or the dynamics of online community participation and peer interactions that illuminate collaborative learning. Future studies should incorporate comprehensive assessments including skills evaluation, qualitative analyses of mentoring relationships, surveys of institutional support structures, and quantitative measures of online engagement to elucidate how these collaborative learning dynamics influence program outcomes. Third, our analysis of participant affiliation was limited to a binary variable (university-affiliated or not), which is an imperfect proxy for the actual research environment. In Japan, university affiliation does not guarantee a supportive research environment [6]. Future studies should collect more granular data on the institutional support environment. Fourth, a challenge remains in the formal assessment of mentees for mentorship roles and the structured faculty development for new mentors. While we provide web-based training on mentoring, a comprehensive evaluation system for these skills is not yet fully established and represents a key area for future development. Fifth, while we provided summary data on publication venues and citations, we did not conduct a formal analysis of article quality metrics beyond indexing status. Future evaluations should include more detailed assessments, such as independent article quality ratings, to better characterize program outputs.

Looking forward, we are exploring large language models as a “third mentor” [26,27] to improve mentor feedback speed and quality while reducing workload. We are investigating the application of large language models in the systematic review process to accelerate research timelines [28,29]. While being mindful of the potential for hallucinations in large language models [30], SRWS-PSG will improve the efficiency of medical scientific endeavors.

### Conclusion

A majority of health care professionals who participated in the SRWS-PSG program submitted manuscripts with low discontinuation rates. This model might address geographical barriers and create a self-sustaining financial structure, providing an adaptable approach for RCB across health care contexts. Future research should incorporate a more comprehensive evaluation of program mechanisms and outcomes.

## Supplementary material

10.2196/78862 Multimedia Appendix 1 Scientific Research Works Peer Support Group (SRWS-PSG) curriculum.

10.2196/78862 Multimedia Appendix 2 Published articles from Scientific Research Works Peer Support Group (SRWS-PSG) mentees.

## Abbreviations

CI: confidence interval
RCB: research capacity building
sdHR: subdistribution hazard ratio
SRWS-PSG: Scientific Research Works Peer Support Group
STROBE: Strengthening the Reporting of Observational Studies in Epidemiology
