# Supplementary material for: Effectiveness of a Fully Online Scientific Research Works Peer Support Group Model for Research Capacity Building Through Conducting Systematic Reviews Among Health Care Professionals: Retrospective Cohort Studies
Source: JMIR Med Educ. 2025 Oct 2;11:e78862. doi: 10.2196/78862 (PMC12490813; doi:10.2196/78862)
Supplement: Multimedia Appendix 1 [file mededu-v11-e78862-s001.docx]

## SRWS-PSG Curriculum

| Category | Serial | Content | Movie time (min:sec) |
| --- | --- | --- | --- |
| Orientation | 01_00 | Practice operations | 0 |
| Orientation | 01_01 | Orientation | 23:38 |
| Orientation | 01_02 | Environment setup | 17:14 |
| Orientation | 01_03 | Structuring clinical questions: For those who don’t know where to start | 17:41 |
| Orientation | 01_04 | Commenting and editing rules using Google document | 0 |
| Orientation | 01_05 | (Extra edition) Let’s keep research records | 9:58 |
| Orientation | 01_06 | Tips for information gathering as lifelong learning | 0 |
| Orientation | 01_07 | “Time management techniques for a stay-at-home mom working mainly in clinical practice to achieve RCT” | 68:26 |
| Orientation | 01_08 | Factors and countermeasures for procrastination | 19:39 |
| Orientation | 01_09 | How to write conclusions in intervention reviews | 8:09 |
| Orientation | 01_10 | Solving the troublesome parts of paper writing with large language models! How to write papers efficiently using ChatGPT | 0 |
| How to write letters | 02_01 | How to write letters | 22:38 |
| How to write letters | 02_02 | Letter to a letter | 0 |
| How to write letters | 02_03 | Handling rejection | 0 |
| How to write letters | 02_04 | Creating a cover letter | 0 |
| How to write letters | 02_05 | How to find letter candidates | 0 |
| How to write letters | 02_06 | Tips for writing letters | 0 |
| Until SRWS Protocol Registration | 03_01 | What can be done with SR | 12:14 |
| Until SRWS Protocol Registration | 03_02 | Research examples | 8:32 |
| Until SRWS Protocol Registration | 03_03 | Searching for previous reviews | 17:06 |
| Until SRWS Protocol Registration | 03_03_1 | For mentees who want to do reviews other than interventions (for mentees whose RQ is not yet decided) | 0 |
| Until SRWS Protocol Registration | 03_04 | How to use literature management software | 8:21 |
| Until SRWS Protocol Registration | 03_04_02 | Operations for SR using ENDNOTE for ENDNOTE users | 0 |
| Until SRWS Protocol Registration | 03_05 | Inclusion/Exclusion criteria | 16:53 |
| Until SRWS Protocol Registration | 03_06 | PubMed search formula part 1 | 31:04 |
| Until SRWS Protocol Registration | 03_06 | How to do the PubMed search formula part 1 assignment | 0 |
| Until SRWS Protocol Registration | 03_07 | PubMed search formula part 2 | 11:35 |
| Until SRWS Protocol Registration | 03_07 | How to do the PubMed search formula part 2 assignment | 0 |
| Until SRWS Protocol Registration | 03_09_01 | Quality assessment of individual studies Preparation | 18:04 |
| Until SRWS Protocol Registration | 03_09_02 | Quality assessment of individual studies Practice | 37:18 |
| Until SRWS Protocol Registration | 03_10 | Meta-analysis: Indicators of existence, incidence, and effect | 19:20 |
| Until SRWS Protocol Registration | 03_11 | Meta-analysis: Heterogeneity and publication bias | 20:11 |
| Until SRWS Protocol Registration | 03_11_1 | Meta-analysis of continuous variables | 18:40 |
| Until SRWS Protocol Registration | 03_12 | Qualitative synthesis stratification_Sensitivity analysis consideration and handling missing data | 14:30 |
| Until SRWS Protocol Registration | 03_12_01 | Practice of subgroup analysis | 11:26 |
| Until SRWS Protocol Registration | 03_13_1 | SoF GRADE PROSPERO registration PRISMA | 13:51 |
| Until SRWS Protocol Registration | 03_13_2 | SoF GRADE PROSPERO registration PRISMA | 24:49 |
| Until SRWS Protocol Registration | 03_13_4 | PRISMA Protocol Registration | 3:29 |
| Until SRWS Protocol Registration | 03_13_5 | Explanation material for the threshold of imprecision in the GRADE approach | 0 |
| Until SRWS Protocol Registration | 03_14 | How to write the background of SR | 15:28 |
| Until SRWS Protocol Registration | 03_14_1 | Paragraph writing | 0 |
| Until SRWS Protocol Registration | 03_15 | Search formulas for other databases | 0 |
| Until SRWS Protocol Registration | 03_15_2 | PRISMA-S | 0 |
| Until SRWS Protocol Registration | 03_16 | SR Protocol Registration on OSF | 3:58 |
| Until SRWS Protocol Registration | 03_17 | Explanation of qualitative synthesis | 0 |
| Until SRWS Results | 04_01 | From protocol to results | 0 |
| Until SRWS Results | 04_02 | Preparation and practice of full-text screening | 0 |
| Until SRWS Results | 04_03 | Efficient inquiries - Inquiries using Word’s mail merge function | 0 |
| Until SRWS Results | 04_04 | Data processing of clinical trial registration sites | 0 |
| Until SRWS Results | 04_05 | How to use GRADEpro GDT | 4:11 |
| Until SRWS Results | 04_06 | How to use Rob2 macro form | 8:23 |
| Until SRWS Results | 04_07 | Before creating a PRISMA flowchart | 0 |
| Until SRWS Results | 04_08 | Removing duplicates from search results | 3:49 |
| Until SRWS Results | 04_09 | About data extraction | 19:30 |
| Until SRWS Results | 04_10 | Search update | 0 |
| Until SRWS Results | 04_11 | Creating Embase search formulas | 0 |
| Until SRWS Results | 04_12 | How to use Rayyan 2024 | 0 |
| From SRWS Results to Submission | 05_01 | From results to submission | 0 |
| From SRWS Results to Submission | 05_02 | Sharing Word files using OneDrive | 2:51 |
| From SRWS Results to Submission | 05_03 | PRISMA 2020 statement (for beginners) | 16:50 |
| When SRWS Revision Comes Back | 06_01 | When revision comes back | 0 |
| Other Lectures | 09_01 | New PubMed search method | 5:14 |
| Other Lectures | 09_02 | SPH writing seminar | 0 |
| Advanced SR Course | 10_01 | Manual for prognosis SR | 0 |
| Advanced SR Course | 10_01_02 | Quality assessment of prognosis research_QUIPS | 0 |
| Advanced SR Course | 10_01_03 | GRADE approach for evaluating evidence of prognostic factors (non-contextualized) | 0 |
| Advanced SR Course | 10_01_04 | GRADE approach for evaluating overall prognosis evidence | 0 |
| Advanced SR Course | 10_02_00 | Pitfalls in interpreting diagnostic accuracy studies | 0 |
| Advanced SR Course | 10_02_01 | Basics of systematic reviews of diagnostic accuracy | 23:25 |
| Advanced SR Course | 10_02_02 | Quality assessment of diagnostic accuracy studies_QUADAS-2_Overview | 12:20 |
| Advanced SR Course | 10_02_03 | Quality assessment of diagnostic accuracy studies_QUADAS-2_Details | 13:21 |
| Advanced SR Course | 10_02_04 | MA in DTASR_Part 1_Description of DTASR results | 27:44 |
| Advanced SR Course | 10_02_05 | MA in DTASR_Part 2_Hierarchical model | 19:49 |
| Advanced SR Course | 10_02_06 | How to summarize DTASR results (for SR) | 0 |
| Advanced SR Course | 10_02_06_02 | How to summarize DTASR results (for guideline creation) | 0 |
| Advanced SR Course | 10_02_07 | QUADAS-C | 8:43 |
| Advanced SR Course | 10_03_01 | Basics of SR incorporating non-randomized studies | 11:32 |
| Advanced SR Course | 10_03_02 | Bias assessment of non-randomized studies_ROBINS-I_Preparation | 19:24 |
| Advanced SR Course | 10_03_03 | Bias assessment of non-randomized studies_ROBINS-I_Practice | 24:51 |
| Advanced SR Course | 10_03_04 | Manual for SR incorporating non-randomized studies | 0 |
| Advanced SR Course | 10_05 | Basics of individual patient data meta-analysis | 72:31 |
| Advanced SR Course | 10_06 | Scoping review | 13:39 |
| Advanced SR Course | 10_07 | Method of Meta-Analysis incorporating Randomized cross-over trials | 0 |
| Advanced SR Course | 10_08 | Prevalence SR | 2:20 |
| Advanced SR Course | 10_10_01 | GRADE for clinical practice guidelines: Certainty assessment of individual outcomes | 0 |
| Advanced SR Course | 10_11_01 | NMA Theory Part 1 | 20:21 |
| Advanced SR Course | 10_11_02 | NMA Theory Part 2 | 12:32 |
| Advanced SR Course | 10_11_03 | NMA Practice | 25:20 |
| Advanced SR Course | 10_11_04 | Network Meta-Analysis Troubleshooting | 0 |
| Advanced SR Course | 10_12 | DTA NMA | 0 |
| Advanced SR Course | 10_13 | Dose-response meta-analysis | 62:45 |
| Observational Study Course | 20_00_01 | Learning from predecessors_Part 1, Part 2 | 0 |
| Observational Study Course | 20_00_02 | Learning from predecessors_Part 3 | 0 |
| Observational Study Course | 20_00_03 | Conceptuarization | 7:00 |
| Observational Study Course | 20_00_04 | Designing measurements | 4:58 |
| Observational Study Course | 20_00_05 | Choosing the study design | 14:45 |
| Observational Study Course | 20_00_06 | Improving the quality of comparison | 11:33 |
| Observational Study Course | 20_00_07 | Ethical considerations | 0 |
| Observational Study Course | 20_01 | Data cleaning and Table 1 | 0 |
| Observational Study Course | 20_02 | Questionnaire survey | 17:54 |
| Observational Study Course | 20_03 | How to write case reports | 35:49 |
| Observational Study Course | 20_03_01 | How any resident can get a case report accepted within a year | 61:25 |
| Observational Study Course | 20_04_01 | Ecological studies using open data | 0 |
| Observational Study Course | 20_05_01 | Framework of diagnostic accuracy studies | 30:30 |
| Observational Study Course | 20_05_02 | Outcome calculation methods for diagnostic accuracy studies | 0 |
| Observational Study Course | 20_06 | Quasi-experimental design | 0 |
| Observational Study Course | 20_07 | Let’s try using DWH | 0 |
| Observational Study Course | 20_07_01 | How to create anonymized IDs | 0 |
| Observational Study Course | 20_08 | Let’s create prediction indices! | 0 |
| Observational Study Course | 20_11 | Meta-epidemiological studies | 10:00 |
| Observational Study Course | 20_12 | Paper evaluation tools used in meta-epi (SR, CPG) | 15:46 |
| Observational Study Course | 20_13 | Research protocol template for ecological studies | 0 |
| Observational Study Course | 20_14 | Let’s create AI! | 0 |
| Observational Study Course | 20_15 | Validation studies for database research | 9:40 |
| Observational Study Course | 20_15 | How to create figures and tables | 0 |
| Observational Study Course | 20_16 | Registering achievements | 0 |
| Observational Study Course | 20_17 | After learning from predecessors | 0 |
| Observational Study Course | 20_18 | Bias and confounding | 6:01 |
| Observational Study Course | 20_19 | Ethics training | 0 |
| Observational Study Course | 20_20 | How to create visual abstracts | 60:17 |
| Observational Study Course | 20_21 | Obtaining literature at facilities without a library | 3:13 |
| Observational Study Course | 20_22 | Let’s try analyzing the receipts from our own facility | 19:04 |
| Mentoring | 40_01 | Research mentoring that no one taught me | 12:06 |
| Mentoring | 40_02 | Individual career plan | 0 |
| Basic Clinical Research Problem Set | 50 |  | 0 |
| Other TIPS | 60_01 | Accelerating questionnaire research with GAS: Exploring uses of ChatGPT other than paper creation | 54:00 |
| Other TIPS | 60_02 | Submission to Cureus | 19:24 |
| Other TIPS | 60_03 | Introduction to image generation AI | 5:25 |
| Other TIPS | 60_04 | New way to write medical English papers using ChatGPT and mentors: Efficient and high-quality paper writing for anyone | 12:34 |
